# Pneumonia in Pregnancy

**DOI:** 10.1155/S1064744995000202

**Published:** 1995

**Authors:** Maurizio Maccato

**Affiliations:** Departments of Pediatrics and Obstetrics and Gynecology Baylor College of Medicine 7400 Fannin #700 Houston TX 77054 USA

## Abstract

Pneumonia complicating pregnancy requires a prompt diagnosis and the institution of adequate supportive and antimicrobial therapy. In a patient with a classic presentation of pneumonia, the most likely pathogens are *Streptococcus pneumoniae* and *Haemophilus influenzae*. In a patient with an atypical presentation of pneumonia, *Mycoplasma pneumoniae* and *Chlamydia pneumoniae* are frequently encountered. In a patient suffering from acquired immunodeficiency syndrome (AIDS), *Pneumocystis carinii* is the most frequent pathogen. The antimicrobial therapy, therefore, has to be tailored to the sensitivity patterns of these pathogens in the community. Hospitalization is recommended for the pregnant patient diagnosed with pneumonia to ensure effective supportive care and minimize the risk of preterm labor and delivery.


Infectious Diseases in Obstetrics and Gynecology 2:283–288 (1995)
© 1995 Wiley-Liss, Inc.

### Pneumonia in Pregnancy

**Maurizio Maccato**

*Departments of Pediatrics and Obstetrics and Gynecology, Baylor College of Medicine, Houston, TX*

ABSTRACT

**Pneumonia complicating pregnancy requires a prompt diagnosis and the institution of adequate supportive and antimicrobial therapy. In a patient with a classic presentation of pneumonia, the most likely pathogens are *Streptococcus pneumoniae* and *Haemophilus influenzae*. In a patient with an atypical presentation of pneumonia, *Mycoplasma pneumoniae* and *Chlamydia pneumoniae* are frequently encountered. In a patient suffering from acquired immunodeficiency syndrome (AIDS), *Pneumocystis carinii* is the most frequent pathogen. The antimicrobial therapy, therefore, has to be tailored to the sensitivity patterns of these pathogens in the community. Hospitalization is recommended for the pregnant patient diagnosed with pneumonia to ensure effective supportive care and minimize the risk of preterm labor and delivery.** © 1995 Wiley-Liss, Inc.

KEY WORDS

Respiratory distress, infection, preterm labor, antibiotic therapy

Pneumonia, the inflammation of alveoli and respiratory bronchioles, is a complication of pregnancy that to this date is associated with significant risk to both the mother and the fetus. In spite of effective antimicrobial therapy, preterm labor and both maternal and fetal mortality remain significant risks, demanding prompt and effective therapy of the condition. Pneumonia can be caused by a variety of both infectious and noninfectious agents. Bacteria and viruses are the most frequent and clinically important infectious etiologies, while parasitic and fungal pneumonias are usually limited to immunocompromised hosts. Aspiration remains the most common noninfectious cause of pneumonia in clinical practice.

### PATHOGENESIS

Pneumonias caused by infectious agents develop when pathogens gain access to the lower respiratory tract and succeed in overwhelming the anatomic and physiologic defenses of the host.

Particles larger than 10 μm in diameter are trapped in the upper airway, while smaller ones are removed by the ciliated epithelium and phagocytized by the lung macrophages. Both humoral and cellular immune responses play a role in the prevention and response to pneumonia. The humoral immune response is mediated by both IgA and IgG antibodies. IgA antibodies are present in the secretions and IgG antibodies can be found in the alveoli and the lung parenchyma. The cellular components of the immune system are more effective once opsonization of the antigens has been achieved.

The infiltration of polymorphonuclear leukocytes that occurs when the infection is established leads to an increase in the volume of alveolar and interstitial fluids and produces a pattern of density on chest radiograph that helps in the evaluation of the pneumonia. Three basic chest radiographic abnormalities have been described: 1) "Air-space" pneumonia is characterized by consolidated lung areas secondary to the exudate present in the alveoli, with relatively uninvolved bronchi leading to

Address correspondence/reprint requests to Dr. Maurizio Maccato, 7400 Fannin #700, Houston, TX 77054.

Received February 17, 1994

Accepted May 24, 1994

Review Article air bronchograms. 2) "Interstitial" pneumonia is characterized by the predominant inflammatory response in the alveolar septa, increasing the interstitial markings and producing a reticular pattern. 3) "Bronchopneumonia" occurs when an air-space pneumonia involves the bronchi themselves leading to atelectasis and to the absence of air bronchograms.

Several predisposing factors to the development of pneumonia have been identified: 1) Endotracheal tubes and depressed gag or cough reflexes remove the upper airway's ability to protect the lower respiratory tract from colonization. 2) Antecedent viral infections or smoking may compromise the mucociliary escalator. 3) The effectiveness of alveolar macrophages is compromised by alcoholism. 4) Anemia or other chronic diseases may interfere with the host immunologic response. 5) Clearly, patients that suffer from immunodeficiencies or are on immunosuppressive drugs are at an increased risk.

The obstetrical patient is at particular risk for aspiration pneumonia. Aspiration pneumonitis is made more likely by the delay in gastric emptying and the decrease in tone of the gastroesophageal sphincter. Aspiration pneumonia is obviously more common in patients who suffer from clouding of consciousness from any etiology, including seizures, alcoholism, or drug overdose, but general anesthesia is the most significant risk factor. The mortality for aspiration pneumonitis depends on the severity of the injury, and it is estimated that more than 100 maternal deaths/year can be directly ascribed to this complication.[1,2]

### MICROBIOLOGY

Because antimicrobial therapy of pneumonia is best started prior to the knowledge of final culture results, the proper empiric antibiotic therapy must be instituted based on the knowledge of the organisms that are most likely to produce the specific clinical presentation of the pneumonia. Different pathogenic organisms are responsible for the pneumonias that present with the "classic" vs. the "atypical" constellation of signs and symptoms. "Classic" pneumonias have a sudden onset, with fever, chills, and a productive cough. A chest radiograph generally shows consolidation of 1 or more lobes. "Atypical" pneumonia has a slow progression of the condition over several days, with a nonproductive cough, low-grade fever, and generalized malaise. The chest radiograph frequently reveals an interstitial pneumonitis.

The most common organism identified in pregnant patients with bacterial pneumonia is *Streptococcus pneumoniae*. In several reports, this organism is responsible for 30–50% of pneumonias in antepartum patients.[3–5] *S. pneumoniae* is a gram-positive encapsulated diplococcus that causes a classic pneumonia with abrupt onset of fever, chills, rusty sputum, and pleuritic chest pain. The patient frequently can recall the precise time of the onset of symptoms. The disease has a very rapid course and is associated with a chest radiograph revealing air bronchograms and consolidation. Pleural effusions are not uncommon. Leukocytosis and an increase in immature polymorphonuclear cells are the rule. The lung parenchyma is not destroyed in streptococcal pneumonia. *Haemophilus influenzae* has been frequently isolated as well.[3–5] It is a gram-negative coccobacillus that, like the pneumococcus, requires opsonization to be phagocytized. Destruction of pulmonary tissue is rare. The chest radiograph shows air bronchograms and consolidation occurring frequently in the upper lobes of the lungs.

*Klebsiella pneumoniae*, an encapsulated gram-negative rod, has been associated with chronic alcoholism. The chest radiograph is significant for consolidation and air bronchograms frequently involving the upper lobes. Because *K. pneumoniae* is associated with destruction of lung tissue, cavitation and abscess formation may occur.

*Staphylococcus aureus* is a frequent bacterial pathogen in patients recovering from viral pneumonia, especially influenza. Staphylococcal pneumonia has an abrupt onset and a rapid course. Pleuritic chest pain and sputum production are the rule. Frequently, pleural effusion and cavitation develop. Staphylococcal pneumonia may also result from the hematogenous dissemination of the organisms. This may occur in an intravenous (IV) drug abuser, a patient with infected endocarditis, or a patient with a contaminated IV catheter. In such cases, the chest X-ray reveals multiple infiltrates in both lung fields. The patient's condition is usually critical.

*Legionella pneumophila* produces an influenza-like syndrome followed by the development of fever, chills, and a nonproductive cough, generally over 3–4 days. Headache and sore throat are common. The organism causes necrosis of the interalveolar septa leading to fibrosis at the resolution of the disease.

Anaerobic bacteria are usually associated with aspiration pneumonia. The onset of the disease is gradual, and it is usually associated with pleural effusion, cavitation, and putrid sputum.

*Acinetobacter, Serratia,* and *Pseudomonas* play a significant role in immunocompromised patients. A large inoculum of these organisms is needed to establish the infection; therefore, they are of particular concern in intubated or unconscious patients. As in the case of *Staphylococcus aureus* pneumonia, gram-negative organisms may be hematogenously deposited in the lungs from sources in the abdomen or in the pelvis or from septic thrombophlebitis causing a pneumonia involving all lung fields, as demonstrated on chest X-ray by multiple infiltrates.

*Francisella tularensis* is a pleomorphic gram-negative organism that produces a rapid onset of fever, chills, and a nonproductive cough. It is acquired by tick bite or exposure to infected animals. The chest radiograph may show the characteristic oval infiltrate near the hilus of the lung, but more commonly the infiltrate is a bronchopneumonia with hilar lymphadenopathy.

The most common agent responsible for the atypical pneumonias is *Mycoplasma pneumoniae.* The onset is insidious, with a nonproductive cough, low-grade fever, and mildly elevated white blood cell (WBC) count in association with a bronchopneumonia on chest radiograph, generally very impressive in relation to the mild-to-moderate symptoms reported by the patient. In association with *M. pneumoniae* infections, bullous myringitis, cervical lymphadenopathy, and skin rashes have been reported. Approximately 70% of patients with *Mycoplasma* pneumonia are cold-agglutinin positive. The test, however, is not specific, with cold agglutinins detected in all other pneumonias and several other infections. Recovery from *Mycoplasma* pneumonia occurs 1–2 weeks after the onset of symptoms.

*Chlamydia pneumoniae* is another organism that causes atypical pneumonia. A more severe pneumonia is caused by *C. psittaci,* acquired by inhalation of contaminated fecal particles from birds infected with the organisms. Human-to-human transmission has been documented but it is rare. Another pneumonia which affects people working with livestock is caused by *Coxiella burnettii,* frequently causing splenomegaly in association with atypical pneumonia.

Influenza virus (usually type A) is the most common viral pathogen involved in pneumonia. *Staphylococcus aureus* or *Streptococcus pneumoniae* may complicate the picture by causing a bacterial superinfection. Adenovirus, measles, and varicella are other viruses that have been associated with some frequency with the development of pneumonia.

Varicella pneumonia is a significant complication of an otherwise benign disease. The chest radiograph reveals a diffused nodular pattern. It develops 3–6 days after the onset of the vesicular eruption, with cough, dyspnea, hemoptysis, and pleuritic chest pain. In pregnancy, significant mortality is associated with this complication.

In immunocompromised patients, fungal and parasitic pneumonias must be kept in the differential diagnosis. The most common parasitic pneumonia is caused by *Pneumocystis carinii* and it affects approximately 80% of patients with acquired immunodeficiency syndrome (AIDS).[6–8] The patient reports dyspnea and a nonproductive cough in association with tachycardia and tachypnea. A diffuse infiltrate on chest X-ray is the norm, but other patterns (including a normal chest radiograph) have been described. A high degree of suspicion is needed to make an early diagnosis in view of the mildness of symptoms at the onset.

Tuberculosis is another infection that must be kept in the differential diagnosis of pneumonia, especially in immunocompromised patients.[9] Multidrug-resistant tuberculosis outbreaks in recent months have been associated with high mortality rates, compelling physicians to reevaluate control measures, preventive therapy for persons exposed, and treatment modalities. The Centers for Disease Control (CDC)[10,11] has recently published guidelines for dealing with these issues.

Pneumonias caused by fungi are generally very mild unless dissemination of the disease occurs.[12] Organisms that may cause pneumonia include *Coccidioides immitis, Histoplasma capsulatum,* and *Blastomyces dermatitidis. Cryptococcus neoformans* is a rare cause of pneumonia, generally causing an encephalitis.[13]

### EVALUATION AND THERAPY

The treatment of the pregnant patient with pneumonia has to focus on supportive care as well as

**TABLE 1. Suggested therapy of pneumonia in pregnancy**

| | First-line therapy | Alternatives |
|---|---|---|
| "Classic" syndrome | Broad-spectrum penicillins, cephalosporins, or penicillins with beta-lactamase inhibitors | Azythromycin or erythromycin |
| "Atypical" syndrome | Azythromycin | Erythromycin |
| Nosocomial | Aminoglycoside plus antipseudomonal penicillin or penicillin with beta-lactamase inhibitor or third-generation cephalosporins | Imipenem cilastatin |
| Aspiration (superinfection) | Aminoglycoside plus clindamycin or penicillin with beta-lactamase inhibitor | Imipenem cilastatin |
| Influenza A | Amantadine or rimantadine plus inhaled ribavirin | |
| Varicella pneumonia | Acyclovir | |

rapid establishment of effective antimicrobial therapy. It is important to secure sputum and blood specimens for cultures to guide the therapeutic choices after the initiation of empiric therapy.

A Gram's stain of the sputum may provide important information if properly collected and examined. The sample must be representative of the secretions in the alveoli and lower bronchial tree; therefore, if large amounts of epithelial cells are noted in the specimen, the sample may be contaminated with oropharyngeal flora. If polymorphonuclear leukocytes are present and very few epithelial cells are noted, the sample is adequate. If a sputum sample cannot be obtained, in certain clinical conditions the clinician may opt to obtain such samples in a more invasive way by transtracheal aspiration, bronchoscopy, or open-lung biopsy. Such decisions have to be made on a case-by-case basis, balancing the risk of the procedure with the importance of the information that may be obtained by the diagnostic modality. Blood cultures should be routinely obtained because of their high specificity if positive. The role of serology is important in the diagnosis of some of the atypical pneumonias, but frequently requires paired acute and convalescent titers, thus limiting their usefulness in the acute setting.

The antibiotic therapy to be instituted is based on the most likely pathogens and their sensitivity patterns in the community where the pneumonia is occurring. Clearly, it is important to distinguish nosocomial vs. community-acquired pneumonia. Table 1 summarizes some of the recommended empiric antibiotic therapies in pregnancy. Such therapy may be modified on the basis of the culture result and the patient's response to therapy.

The antibiotic treatment of community-acquired, classic-onset pneumonia is generally with broad-spectrum penicillins or cephalosporins. Erythromycin may be an alternative regimen. If the community-acquired pneumonia presents with an atypical syndrome, erythromycin or azythromycin is currently the drug of choice in the pregnant patient. If *L. pneumophila* is suspected, rifampicin has been recommended in addition to erythromycin for seriously ill patients.[14,15] Because of the likelihood of *Staphylococcus aureus* in bacterial superinfections after influenza pneumonia, a first-generation cephalosporin or nafcillin should be used in this setting. If the pneumonia is hospital acquired, above all, in an ICU setting, both aerobic and anaerobic organisms should be considered possible pathogens. Broad-spectrum antimicrobial therapy is then of critical importance. A third-generation cephalosporin in combination with an aminoglycoside is a regimen that has been used successfully.[16–18] If the patient suffers from aspiration pneumonia, IV clindamycin in combination with an aminoglycoside is felt to be superior to penicillin G.[16–18]

Varicella pneumonia should be treated with IV acyclovir at high doses, i.e., 10 mg/kg q 8 h.[19–22] Influenza pneumonia can be treated with inhaled ribavirin and, if influenza A is suspected, amantadine or rimantadine at 100 mg p.o. q 12 h.[23] Of note is that amantadine and rimantadine are teratogenic at high doses in some animal models; therefore, these drugs should be used in pregnancy only when indicated. It is important to note that influenza vaccination is not contraindicated in pregnant women and should be encouraged, especially in the presence of heart, lung, or renal disease. Of critical importance is the need for supportive care of the pregnant patient. Specifically, the prevention of hypoxia by oxygen supplementation and the correction of bronchospasm by the use of bronchodilators are important early steps. We routinely use a pulse oximeter to monitor the condition of the patient and provide oxygen supplementation to maintain oxygen saturation of >95%. Mechanical ventilation is required if respiratory failure develops with $PaO_2$ of <60 mm of mercury or ventilatory failure with increasing $PaCO_2$ and acidosis. Delivery of the fetus may be necessary if the maternal cardiovascular and respiratory functions cannot be properly maintained.

In aspiration pneumonia, the key is prevention. Neutralization of gastric acid, coupled with intubation and extubation techniques that minimize the risk of aspiration, is of crucial importance. A large portion of the lung damage that occurs in aspiration pneumonia is secondary to the damage caused by the pH of the gastric aspirate to the alveolar capillary membrane. It is of rapid onset and may cause extensive damage. The superior segments of the lower lobes are most commonly affected if the aspiration occurs when the patient is supine. The use of steroids or prophylactic antibiotics is controversial, but there is no debate on the need to start prompt, effective antibiotic therapy if bacterial superinfection is suspected. Such a superinfection usually develops 2–3 days after the initial injury. The combination of a third-generation cephalosporin and aminoglycoside is recommended. Again, supportive therapy including bronchodilators and possible mechanical ventilation is indispensable.

### CONCLUSIONS

Pneumonia in the pregnant woman is a serious complication that requires aggressive, prompt intervention. Hospitalization is usually required to ensure the most effective supportive care to the patient and to minimize the risk to mother and fetus. The rapid institution of therapy with appropriate antimicrobial agents on the basis of the most likely pathogens involved will provide the best chance for an uneventful resolution of the infection. Preventive efforts should be maximized, including influenza or pneumococcal vaccination of patients at high risk for contracting the disease and all possible measures to prevent nosocomially acquired pneumonia or aspiration pneumonia.

### REFERENCES

1. Kallos T, Lampe KF, Orkin FK: Pulmonary aspiration of gastric contents. In Orkin FK, Cooperman LH (eds): Complications in Anesthesiology. Philadelphia: J.B. Lippincott, 1983.
2. Morgan M: Anesthetic contribution to maternal mortality. Br J Anaesth 59:842–855, 1987.
3. Benedetti TJ, Valle R, Ledger WJ: Antepartum pneumonia in pregnancy. Am J Obstet Gynecol 144:413–417, 1982.
4. Hopwood HG: Pneumonia in pregnancy. Obstet Gynecol 25:875–879, 1965.
5. Oxorn H: The changing aspects of pneumonia complicating pregnancy. Am J Obstet Gynecol 70:1057–1063, 1955.
6. Minkoff H, De Regt RH, Landesman S, et al.: *Pneumocystis carinii* pneumonia associated with acquired immunodeficiency syndrome in pregnancy: A report of three maternal deaths. Obstet Gynecol 67:284–287, 1986.
7. Rankin JA, Collman R, Daniele RP: Acquired immunodeficiency syndrome and the lung. Chest 94:155–164, 1988.
8. Rogers MF, Ewing EP, Warfield D, et al.: Virologic studies of HTLV-III/LAV in pregnancy: Case report of a woman with AIDS. Obstet Gynecol 68(Suppl 3):2S–6S, 1986.
9. Maccato ML: Pneumonia and pulmonary tuberculosis in pregnancy. Obstet Gynecol Clin North Am 16(2):417–430, 1989.
10. Centers for Disease Control: Guidelines for preventing the transmission of tuberculosis in healthcare settings with a special focus on HIV-related issues. MMWR 39:1–29, 1990.
11. Centers for Disease Control: Management of persons exposed to multidrug-resistant tuberculosis. MMWR 41(RR-11):61–71, 1992.
12. Sarosi GA: Management of fungal diseases. Am Rev Respir Dis 127:250–253, 1983.
13. Silberfarb PM, Sarosi GA, Tosh FE: Cryptococcosis and pregnancy. Am J Obstet Gynecol 112:714, 1972.
14. Fitzgeorge RB, Baskerville A, Featherstone ASR: Treatment of experimental Legionnaire's disease by aerosol administration of rifampicin, ciprofloxacin, and erythromycin. Lancet 1:502–503, 1986.
15. Soper DE, Melone PJ, Conover WB: Legionnaire disease complicating pregnancy. Obstet Gynecol 67(Suppl 3):10S–12S, 1986.
16. Bartlett JG, Gorbach SL: Treatment of aspiration pnemonia and primary lung abscess. Penicillin G vs. clindamycin. JAMA 234:935–937, 1975.
17. Grassi GG: Respiratory infections: Established therapy and its limitations. Clin Ther 7(Suppl A):19–36, 1985.
18. Macfarlane JT: Treatment of lower respiratory infections. Lancet 2:1446–1449, 1987.
19. Eder SE, Apuzzio JJ, Weiss G: Varicella pneumonia during pregnancy. Treatment of two cases with acyclovir. Am J Perinatol 5:16–18, 1988.
20. Hankins GD, Gilstrap LC, Patterson AR: Acyclovir treatment of varicella pneumonia in pregnancy (letter). Crit Care Med 15:336–337, 1987.
21. Landsberger EJ, Harger WD, Grossman JH: Successful management of varicella pneumonia complicating pregnancy. A report of three cases. J Reprod Med 31:311–314, 1986.

22. Straus SE: The management of varicella and zoster infections. Infect Dis North Am 1:367–382, 1987.
23. Kirshon B, Faro S, Zurawin RK, et al.: Favorable outcome after treatment with amantadine and ribavirin in a pregnancy complicated by influenza pneumonia: A case report. J Reprod Med 33:399–401, 1988.
